# A Comparison of Two-Handed Face-Mask Ventilation Techniques by Trainees: A Prospective Observational Study

**DOI:** 10.7759/cureus.81002

**Published:** 2025-03-22

**Authors:** Yulia Obelcz, Adrienne James, Abhijit V Lele, Andrew M Walters

**Affiliations:** 1 Anesthesiology and Pain Medicine, Harborview Medical Center, Seattle, USA; 2 Neurocritical Care/Anesthesiology, Harborview Medical Center, Seattle, USA

**Keywords:** anesthesia, anesthesiology, circumference, face-mask, hand, mask ventilation, novice, operating room, two-handed

## Abstract

Background: Effective face-mask ventilation (FMV) is a crucial step for oxygenation after anesthesia induction and prior to airway instrumentation. Preceding literature demonstrates that specific physical characteristics of providers affect tidal volume (Vt) delivery during FMV. In difficult ventilation scenarios, FMV success rates are improved using two-handed techniques, CE or VE. We hypothesized that anesthesia trainees' Vt delivery efficacy would differ with each technique and related to their hand size.

Methods: In this prospective crossover trial, 38 anesthesia trainees performed FMV on elective surgical patients after induction of general anesthesia with both CE and VE techniques. We recorded differences in delivered Vt with each method and analyzed its relation to multiple trainee hand size measurements, including palm circumference, hand length, and hand span.

Results: Thirty-eight operators (females 55.3%, n=21, males 44.7%, n=17) performed FMV on 38 patients (age 48.1+16.7 years, male sex 65.8%, n=25). Larger median Vts were obtained with VE vs. CE (10.0 (5.8-13.6) mL/kg vs. 11.9 (8.9-14.5) mL/kg, p = 0.008) without a significant change in achieving TV of 4 mL/kg (15% vs. 8%, p=0.32). The differences in VE and CE were inversely proportional to hand measurements (circumference: R-square = 0.15, p=0.02, length: R-square = 0.24, p=0.002, and span: R-square = 0.23, p=0.002). When stratifying by quartile of hand size, significant differences were observed as follows: hand size (circumference: first quartile p=0.039, hand length: first quartile p=0.018, second quartile p=0.028, hand span: second quartile p=0.001).

Conclusions: In novice anesthesiology providers, hand size is correlated with delivered Vt during two-hand FMV. The VE modification increases the delivery of Vt, especially in trainees with smaller hands.

## Introduction

Face-mask ventilation (FMV) is a fundamental skill for health care providers, particularly anesthesiologists who routinely perform FMV during the induction of anesthesia and airway emergencies. Inefficient FMV correlates with a significant mortality increase in critically ill patients [1]. In the operating room, inadequate FMV during induction of anesthesia may cause significant desaturation, especially in patients with decreased functional residual capacity [2]. While basic FMV is easier to learn than orotracheal intubation [3], mastery may require lengthy practice. Creating a face mask seal or overcoming upper airway obstruction may be challenging in some patients. Familiarity with various FMV techniques and the ability to adjust to the individual patient’s needs is a core learning objective for students of airway management.

There are one-handed and two-handed methods of holding a mask during FMV. The one-handed technique allows a solo provider to compress the reservoir bag using the free hand. Two-handed FMV requires assistance in delivering breaths; it augments ventilation when a one-handed technique is insufficient, allowing better support of the airway patency and larger tidal volume (Vt) delivery [4-7]. Two-handed FMV can be applied with either CE or VE techniques [8] based on the masking hands' configuration resemblance to letters of the Latin alphabet. Both methods focus on creating a bilateral mask-face seal with simultaneous jaw thrust, “lifting the face into the mask.” They differ in the positioning of the second to fifth fingers and jaw-lifting force distribution between the fingers.

Prior literature is inconclusive about whether the operator’s physical characteristics add to the FMV learning challenges. In a study by Koga and Kawamoto, female anesthesia residents had more difficulties with one-handed mask ventilation than male peers. Female residents frequently had to switch to the two-handed technique to deliver adequate ventilation [9]. In an earlier study by Thomas et al., male and female providers with small hands delivered significantly lower volumes using a single-handed FMV than their large-hand peers [6]. Hess and Spahr also demonstrated a direct correlation between hand size and volume delivered via simulated bag-mask ventilation [10]. There has been no demonstrable difference between the delivered volume and the provider’s physical characteristics when ventilating with a disposable air-cushioned mask. Still, it may result in increased volumes with male gender and larger hand size with a reusable silicone face mask [11]. And finally, Lee et al. found no correlation between ventilation volumes, provider’s gender, hand size, or grip strength [7].

The primary aim of this study was to establish a correlation, or lack thereof, between hand size and Vt delivered by CE and VE FMV techniques. We tested the hypothesis that a) Vt delivered by CE and VE FMV differ and b) the CE-Vt and VE-Vt delivered may depend on operator hand measurements. The knowledge gained from this study may inform educators and novice operators about the optimal choice of FMV technique that suits the individual anesthetists better in ventilation delivery and supporting oxygenation after anesthetic induction.

## Materials and methods

Institutional review board

This study (STUDY00007484) was reviewed and approved (approval date July 10, 2019) by the Institutional Review Board of the University of Washington.

Study design and study setting

This prospective observational study was conducted at the University of Washington Harborview Medical Centre, an academic Level I trauma and Comprehensive Stroke Centre, between July 10, 2019 and February 15, 2022.

Study participants and study details

Participants

The study included patients undergoing general anesthesia that required FMV and tracheal intubation as part of their anesthetic care. We included patients over the age of 18 years and those with the American Society of Anaesthesiologists Physical Classification I-III undergoing elective surgery. Exclusion criteria were the age of under 18 years, pregnancy, incarceration, limited decision-making capacity, known or predicted difficult mask ventilation, increased aspiration risk, or any condition for which the primary anesthesiology attending felt that mask ventilation would be impractical or impossible. The study coordinator (author AJ) screened daily surgical caseloads for patient eligibility and confirmed the results with the attending anesthesiologist, then approached the patients regarding the study enrolment, providing informed consent to the patients who chose to enroll. The study included novice anesthesia providers (operators) such as anesthesiology residents, emergency medicine interns, and fourth-year medical students with less than one month of anesthesia experience.

Study Protocol

After obtaining informed consent from the patient and the trainee, baseline patient characteristics were recorded: age, sex, height, weight, and ASA physical classification. Before the operation, each participant underwent an independent airway exam by a study investigator.

We obtained the following novice anesthesia provider’s hand measurements: 1) The hand span, defined as the distance between the tip of the thumb and the fifth finger, with the hand spreading as wide as possible [12]. 2) The hand’s length as a distance from the midpoint of the distal wrist crease to the most distal point of the third finger [13]. 3) Palm circumference as the perimeter of the middle section of the hand at the two major transverse palmar creases [14]. All measurements were taken on both hands, and an average was calculated.

*Two Types of FMV* 

**Figure 1:**
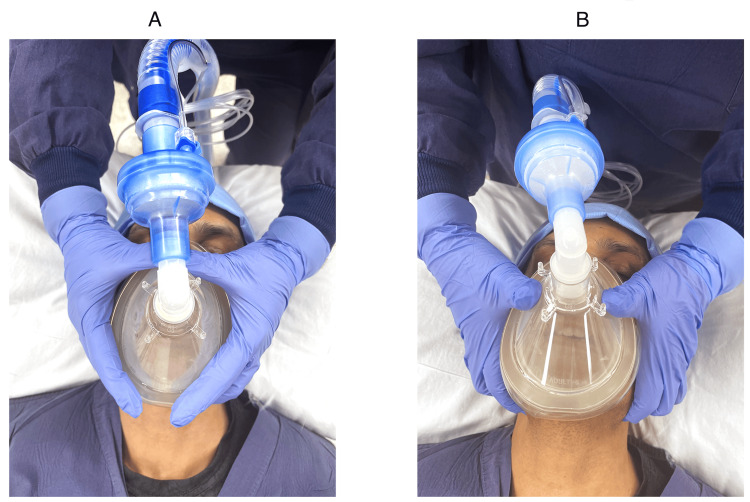
(A) CE and (B) VE techniques of face-mask ventilation Note: Obtained with consent.

In the CE technique, each thumb and index finger form a “C” shape over each side of the mask, keeping the mask firmly on the face (Figure 1A). The three remaining fingers of both hands form the “E” shape, lifting the mandible toward the mask, with the fifth finger often supporting the mandibular angle. CE technique has historically been the most common technique for two-handed FMV and was the only technique described in the literature on airway management [8,15]. The VE (Figure 1B) technique uses the thumbs to hold the mask sealed to the face, with the rest of the hand rotating down at the wrist, as close to 90 degrees as possible. The angle between the thumb and the index finger forms a “V” shape. The second to fifth digits, creating an “E” shape, pull the jaw upward. The VE technique gained popularity after the publication by Joffe et al. [4] and is now included in the airway training literature [16,17].

**Figure 2:**
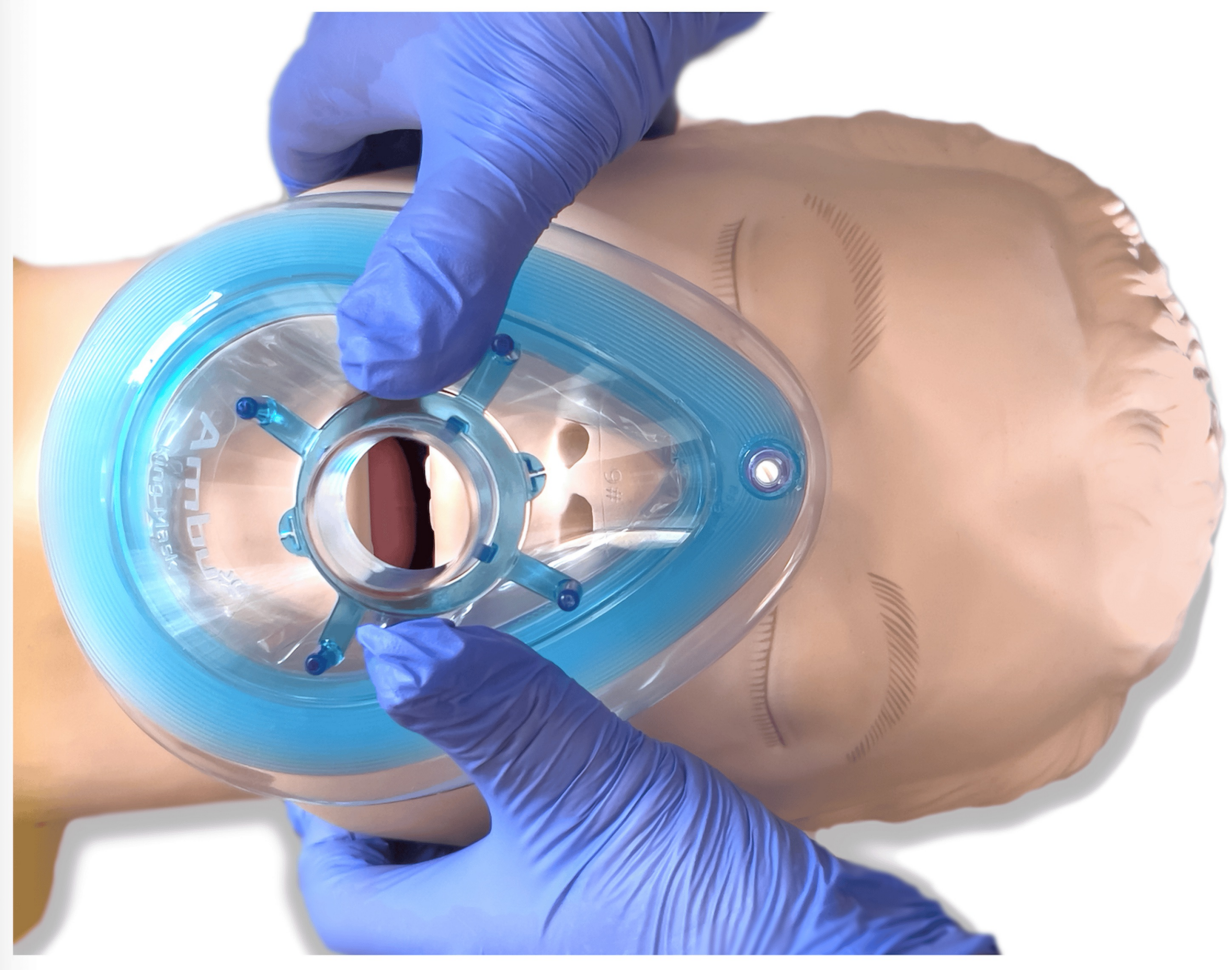
Alternate hand placement in VE technique VE technique with the operator's thumbs directed toward the mask orifice. Only a small percentage of study participants were able to position their hands in this manner.

With the VE masking technique, the length of the thumbs in some individuals allows for the thumb placement not along the side of the mask but directed toward the mask orifice (Figure 2).

In the operating room, each patient had standard ASA monitors applied (electrocardiography, pulse oximetry, non-invasive blood pressure, end-tidal carbon dioxide, and respiratory rate). Preoxygenation with 100% oxygen for two to three minutes was followed by general anesthesia induction with fentanyl (1-2 mcg/kg), propofol (2-3 mg/kg), and rocuronium (0.6 mg/kg). The attending anesthesiologist performed FMV with the one-handed technique until the state of complete muscle paralysis (train of four counts of zero) was confirmed by a quantitative neuromuscular monitoring device. Any patient who required an oropharyngeal airway or two-handed mask ventilation during this time was excluded from the study. Upon confirmation of complete muscle paralysis, the investigator instructed the trainee to insert an oropharyngeal airway into the patient’s airway and apply any two-handed FMV technique. An oropharyngeal airway was utilized to mirror typical clinical practice, where it is generally inserted before employing two-handed FMV in patients who exhibit insufficient ventilation with the single-hand technique. At the same time, the investigator instituted the ventilation by the anesthesia machine (GE Avance CS2 (GE Healthcare, Karnataka, India) in pressure control mode with a respiratory rate of 10 bpm, inspiratory pressure of 15 cmH_^2^_O positive, and positive end-expiratory pressure (PEEP) of 5 cmH_2_O. The trainee maintained the patient’s airway with a two-handed technique of choice for the entire duration of ten respiratory cycles. After completing the first ten respiratory cycles, the investigator briefly demonstrated the alternative FMV technique and instructed the trainee to use that technique for another 10 respiratory cycles. During the study protocol, we recorded the patients’ oxygen saturation, heart rate, blood pressure, and expired Vt for each breath as measured by the anesthesia machine spirometer. After the second cycle of 10 respirations, the primary anesthesia team resumed the planned patient’s care. With this protocol, each patient and each trainee served as their control group. The study supervising investigators (authors YO, AVL, and AW) were blinded to the hand measurement data, and the novice anesthesiology provider was blinded to the Vt data.

Data Collection

In patients, baseline sample characteristics included age, sex, weight, body mass index, and vital signs, including oxygen saturation and Vt. In novice anesthesiology providers, the factors included sex and hand measurements expressed as mean+SD.

We recorded the delivered VE and CE Vt in mL and expressed them as medians. We also compared the delivered Vt to the benchmarked Vt of 4 mL/kg ideal body weight for each patient, as described in prior studies [4]. 

Statistical analysis

The hand measurements, specifically the base of the thumb to the tip of the fifth finger length (TP), the base of the palm to the tip of the middle finger (PMF) length, and the hand circumference (CIRC), were used as stratification variables. Each measurement was divided into quartiles to categorize subjects based on these hand measurements. Quartiles were created using the “quantile” function in R, assigning each measurement into four groups labeled Q1, Q2, Q3, and Q4. Box plots were generated to visualize the distribution of the difference between VE and CE across the quartiles of each hand measurement. Each box plot included a reference line at zero on the Y-axis to indicate no difference between VE and CE. The analysis was conducted for three hand measurements: TP, PMF, and CIRC. The box plots were created using the base R plotting functions. The median difference between VE and CE was calculated for each quartile of the hand measurements to compare the medians of VE and CE. To test for significant differences in the median VE-CE difference across the quartiles of each hand measurement (TP, PMF, and CIRC), a non-parametric “Kruskal.test” function in R, which performs the Kruskal-Wallis test, was used. This non-parametric test compares the medians of two or more independent groups and is appropriate when the assumptions of the ANOVA (such as normality) are not met. The Kruskal-Wallis test was applied to the VE-CE differences across the quartiles for each hand measurement.

Regression analyses assessed the relationship between VE and CE and each hand measurement quartile, stratified by the provider's sex. The regression models included interaction terms between the hand measurement quartiles and sex. The results were visualized using regression plots with 95% confidence intervals. We reported the results as R-square and p-values, and the fitted graphs displayed 95% confidence intervals. Python (Python Software Foundation, Wilmington, DL) and R studio 1.554 (Posit PBC, Boston, MA) (www.rstudio.com) were used for statistical analysis, and a two-tailed p-value of < 0.05 indicated statistical significance.

## Results

Patient characteristics

Baseline characteristics of the 38 study patients are presented in Table 1.

**Table 1:** Patient characteristics ASA: American Society of Anesthesiologists; SD: standard deviation; kg: kilograms: Ideal body weight: ideal body weight of the patients

Characteristics	n=38
Age (years) mean+SD	48.1+16.7
Male sex, n(%)	25(65.8)
ASA classification, n(%)	
ASA Class I	1(2.6)
ASA Class II	25(65.8)
ASA Class III	12(31.6)
Presence of facial hair, n(%)	12(31.6)
Type of airway device used	
Oropharyngeal	37(97.4)
Nasopharyngeal	1(2.6)
Ideal body weight (kg), mean+SD	53.6+10.6

Patients were 48+16.7 years of age, males (66%, n=25), with an average BMI of 28+5 kg/m^2^, of ASA class I (2.6%, n=10), ASA class III (65.8%, n=25), and ASA class III (31.6%, n=12), and 32% (n=12) with facial hair.

Novice anesthesiology provider characteristics

Thirty-eight operators (females, 55.3%, n=21, males, 44.7%, n=17) participated in this study. The mean thumb-to-fifth finder measurement was higher in males than in their female counterparts (8.52+0.7 cm vs. 7.7+0.6 cm, p<0.001). The mean palm-to-middle finger measurement was higher in males than in their female counterparts (7.8+0.5 cm vs. 7.2+0.4 cm, p=0.0001. The mean palm circumference was also higher in males than in females (8.4+0.6 cm vs. 7.4+0.6 cm, p<0.001). The baseline characteristics of the novice providers are presented in Table 2.

**Table 2:** Novice anesthesiology provider characteristics SD: standard deviation; n: counts

Characteristics	n=38
Female sex, n(%)	21(55.3)
Hand circumference (cm), mean+SD	7.85+0.8
Thumb to fifth finger measurement (cm), mean+SD	8.11+0.8
Palm to middle finger measurement (cm), mean+SD	7.46+0.6

Difference between VE and CE techniques by hand measurements

Significant differences in VE-CE were observed across TP quartiles (test statistic = 14.4149, p=0.0024), indicating the superiority of VE in certain quartiles. Differences in VE-CE were also significant across PMF quartiles (test statistic = 11.7652, p=0.0082), suggesting VE's superior performance in certain quartiles. No significant differences in VE-CE were found across CIRC quartiles (test statistic = 7.6393, p=0.0541), indicating no clear superiority of VE based on CIRC quartiles. The results are presented in Table 3.

**Table 3:** The differences in medians of VE (ideal body weight) and CE (ideal body weight) tidal volumes by hand measurement quartiles Note: The differences were evaluated using the Kruskal-Wallis test.

Group	Test statistic	P-value
Base of thumb to tip of the fifth finger	14.4148886	0.00239151
Base of palm to the top of the middle finger	11.7651968	0.00823234
Hand circumference	7.63926031	0.05408618

**Figure 3:**
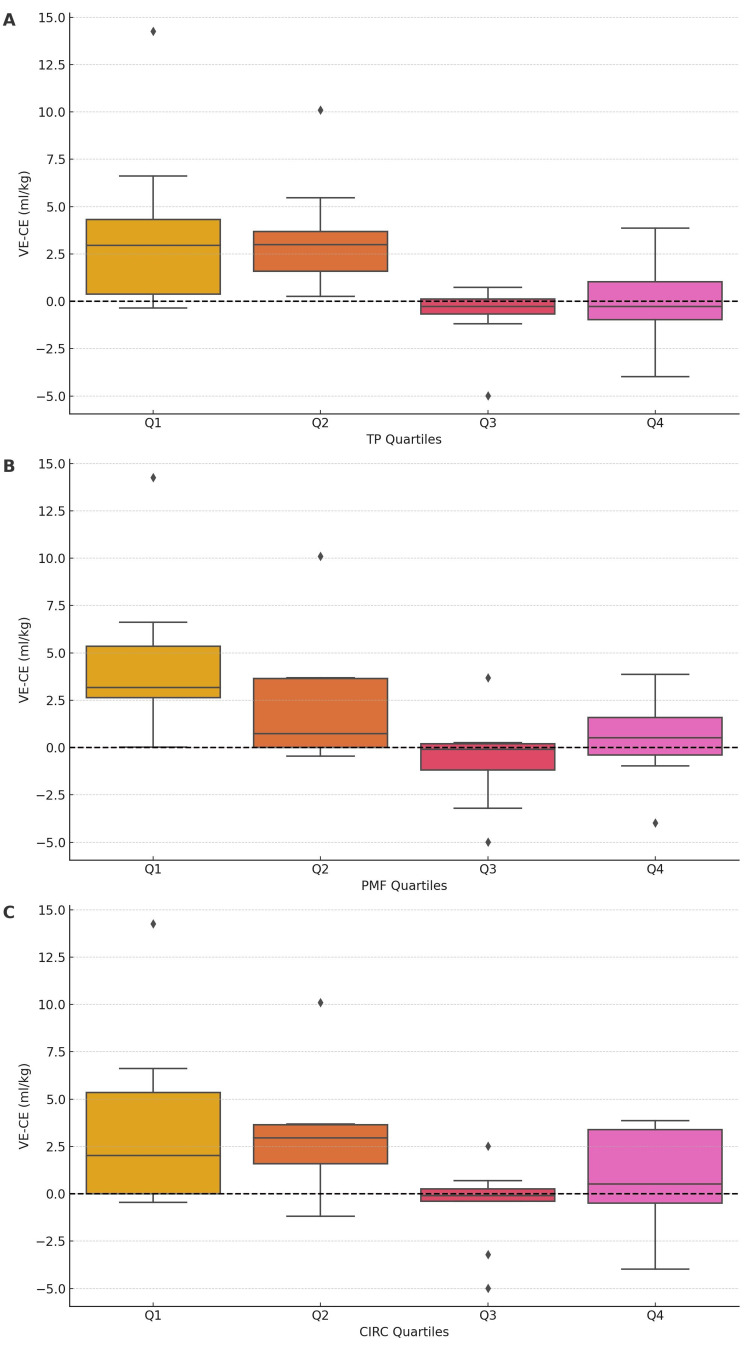
The differences in medians of VE (ideal body weight) and CE (ideal body weight) by hand measurement quartiles TP: Base of thumb to tip of the fifth finger; PMF: Base of palm to the top of the middle finger; CIRC: Hand circumference Note: Y-axis: mL/kg ideal body weight, X-axis: Q1: first quartile, Q2: second quartile; Q3: third quartile; Q4: fourth quartile (A) Difference in tidal volume achieved by VE and CE technique by TP quartiles. (B) Difference in tidal volume achieved by VE and CE technique by PMF quartiles. (C) Difference in tidal volume achieved by VE and CE technique by CIRC quartiles.

The corresponding box plots are shown in Figures 3A-3C.

**Figure 4:**
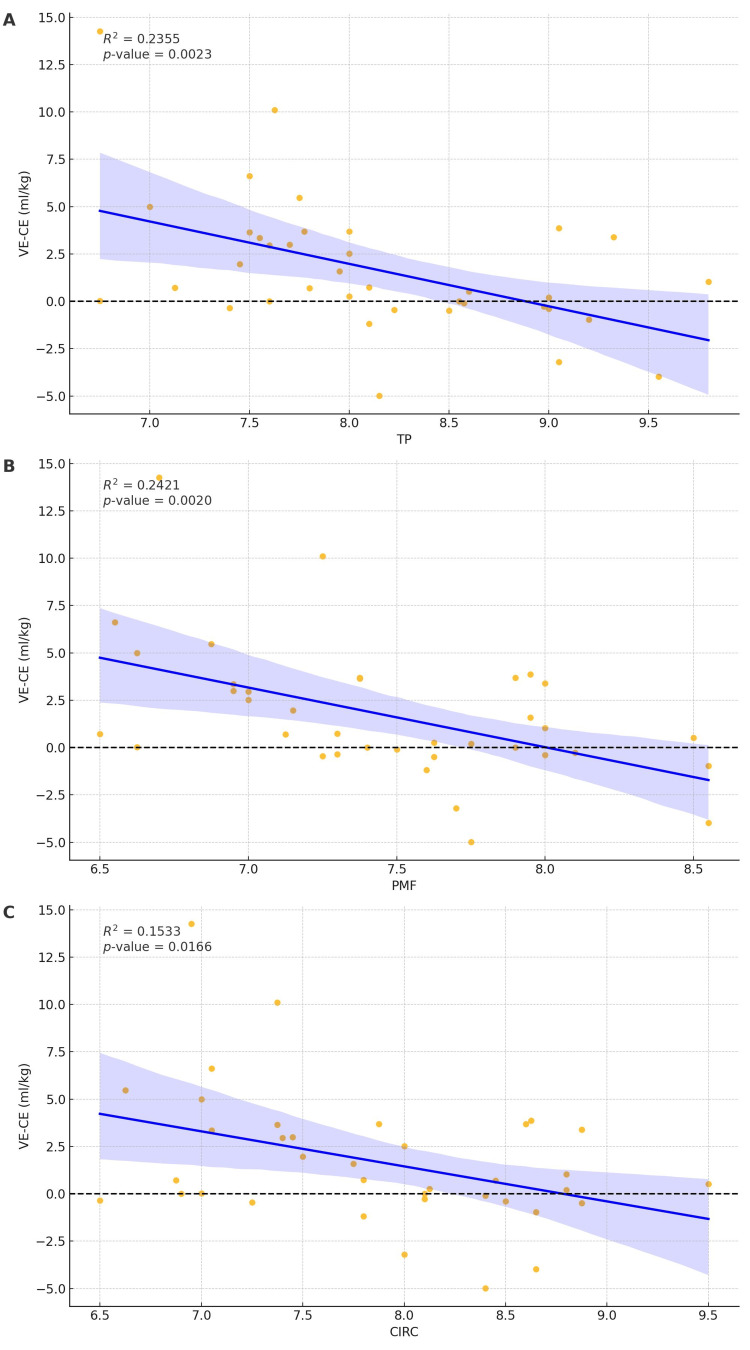
Regression plots of difference in tidal volumes by VE and CE technique and by hand measurements TP: Base of thumb to tip of the fifth finger; PMF: Base of palm to the top of the middle finger; CIRC: Hand circumference (A) Regression plot of the difference in tidal volumes achieved by VE and CE technique by TP measurement. (B) Regression plot of the difference in tidal volumes achieved by VE and CE technique by PMF measurement. (C) Regression plot of the difference in tidal volumes achieved by VE and CE technique by CIRC measurement.

Regression plots of difference in VE and CE by hand measurements (Figures 4A-4C).

**Figure 5:**
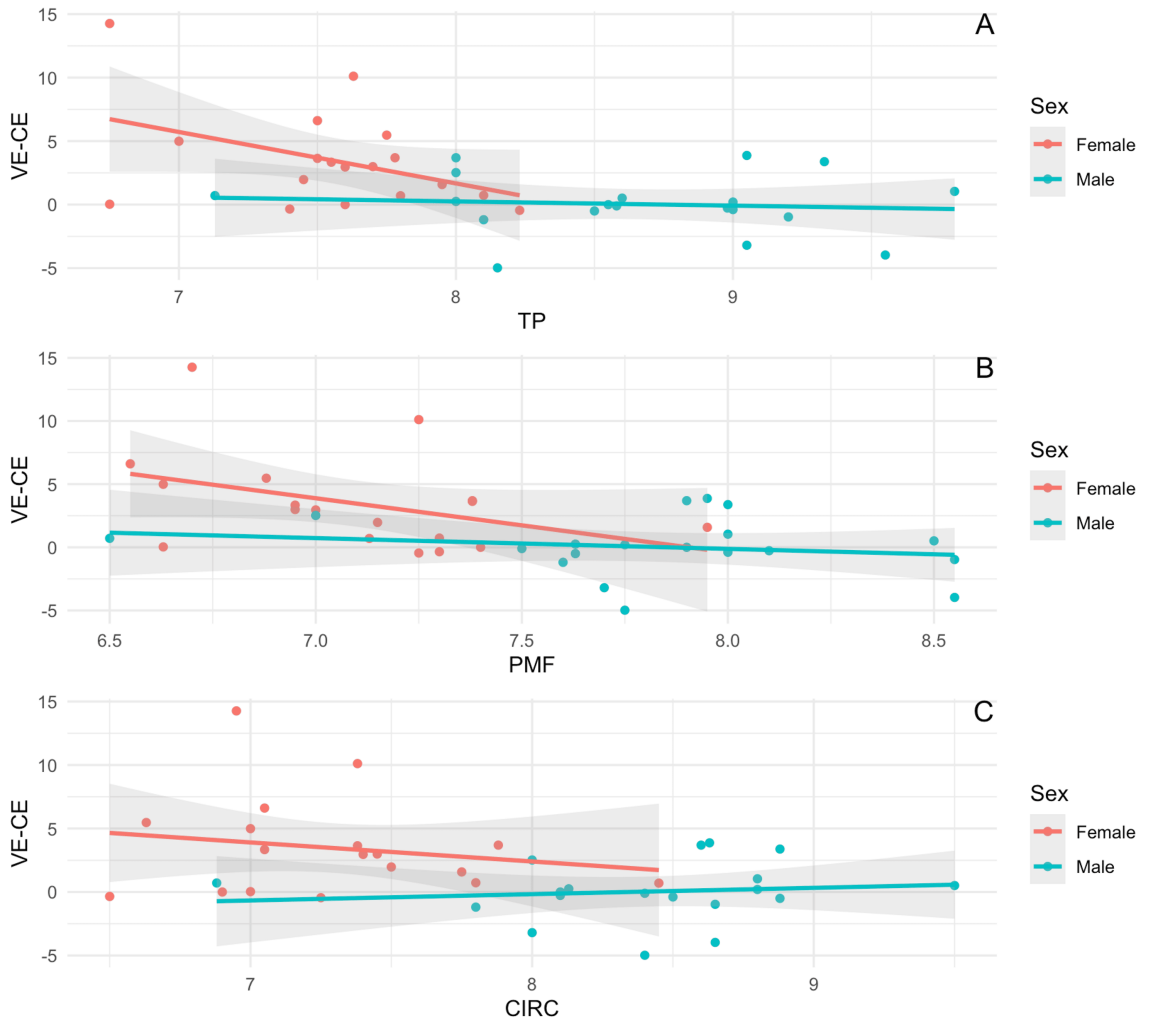
Regression plots by sex of the anesthesiology trainee TP: Base of thumb to tip of the fifth finger; PMF: Base of palm to the top of the middle finger; CIRC: Hand circumference The X-axis measurements are in cm. The Y-axis is in mL/kg. (A) This panel displays the relationship between TP and the difference in tidal volumes achieved by the VE and CE techniques across subjects. (B) This panel displays the relationship between PMF and the difference in tidal volumes achieved by the VE and CE techniques across subjects. (C) This panel displays the relationship between CIRC and the difference in tidal volumes achieved by the VE and CE technique across subjects.

The regression analysis results show a negative correlation between increasing hand measurement and the difference between VE and CE. Specifically, when the distance between the base of the thumb and the tip of the fifth finger increases, there is a decrease in VE-CE (R^2^ = 0.235, p=0.0023). Similarly, when there is an increase in distance between the base of the palm and the tip of the fifth finger, there is a reduction in VE-CE (R^2^=0.2420, p=0.0020), and when hand circumference increases, there is a reduction in the difference between VE and CE (R^2^=0.1533, p=0.0166). Regression plots by sex of the anesthesiology trainee (Figures 5A-5C).

We did not find a statistically significant correlation between VE and CE by hand measurements when data were stratified by sex. These indifferences were across males (TP: R^2^ 0.0086, p=0.7056, PMF: R^2^ 0.0314, p=0.4681, CIRC: R^2^ 0.0129, p=0.6437), and females (TP: R^2 ^0.1789, p=0.08, PMF: R^2^ 0.1531, p=0.1084, CIRC: R^2^ 0.0352, p=0.456).

## Discussion

We conducted this prospective observational study to examine the correlation between trainee hand size and trainee ability to effectively ventilate an anesthetized patient using different two-handed FMV techniques (CE and VE). We ranked the effectiveness based on the ability to deliver Vt >4 mL/kg ideal body weight. We recorded and compared differences in Vt achieved with both CE and VE techniques. The study's primary results were 1) the number of breaths with Vt >4 mL/kg ideal body weight was significantly higher in the VE than in the CE technique and 2) the differences in Vt between CE and VE FMV techniques depended on the operator’s hand measurements, particularly the hand circumference. Operators with smaller hands could achieve larger Vt utilizing the VE technique but not the CE technique.

The differences in Vt between the techniques may be attributable to the following factors. CE technique allows for somewhat limited hand and finger movement once the grip of the mask and the face has been established. One can “lift the face into the mask” by upward wrist rotation, but the position of the fingers on the patient’s face is fixed. Additionally, both fifth digits must be employed in the face-lifting motion. With the smaller hands, the length and/or the strength of the fifth digits and wrist muscles may not be sufficient for a successful lifting maneuver. With the VE technique, more of the forearm and arm muscles may be utilized to perform jaw thrust, allowing the smaller hands to compensate for the smaller size and/or strength. In addition, with the VE technique, the thumbs could be moved along the mask, allowing for dynamic adjustments of the mask to the face. The fact that VE-Vt was significantly higher than CE-Vt could be crucial in assuring proper ventilation and oxygenation, particularly in the challenging mask ventilation scenarios.

The study's finding of a correlation between Vt in CE and VE FMV techniques and the size of the operator's hand may be significant in the field of teaching airway management. The high percentage of female operators in the study (55.3%) presents an intriguing point for discussion, especially in the context of how gender-related physical attributes might influence FMV technique and effectiveness. Here are some key aspects to consider. There are observable differences in hand size and strength between genders. Women tend to have smaller hands [18] and less grip strength compared to men. In the context of FMV, where creating a seal and applying the correct pressure is crucial, these differences might impact the technique and effectiveness. As suggested by the study, smaller hand sizes might lead to challenges in maintaining an adequate mask seal or could necessitate different techniques to achieve the same ventilation efficacy. Providers with smaller hand sizes may naturally adapt their techniques to compensate for hand size and strength differences. This could involve using different hand positions, leveraging body weight differently, or employing alternative strategies to ensure adequate ventilation. These adaptations might influence the effectiveness of FMV and the operator's comfort and endurance during prolonged procedures. The study's high percentage of female operators underscores the need for inclusive training that considers physical differences among healthcare providers. With the increasing representation of female physicians in surgery and anesthesiology [19,20], several providers with smaller hands are also expected to grow. This demographic shift may bear potential implications for equipment manufacturing (e.g., surgeons with small hands are ergonomically disadvantaged when handling specific surgical instruments [21,22]. Training programs might need to offer techniques adaptable to different body types and physical strengths, ensuring all practitioners can perform FMV effectively. 

Limitations

A limitation of this study is that patients who were either anticipated to be difficult to mask or who were discovered to be difficult to mask prior to the study protocol's start were excluded. The VE masking technique is more efficient in patients with a body mass index of 25-35 kg/m^2^ [23], but the data related to hand size is lacking. While our study suggests novice trainees with smaller hand sizes would likely perform better in this population, they could have had equal difficulty with either technique. We included only anesthesia trainees inexperienced at both methods, and these results may not apply to experienced providers. We did not include thumb length and grip strength in the trainee characteristics. Another study may be needed to evaluate the correlation between these two parameters and the effectiveness of different FMV techniques. Longitudinal studies that track the development of FMV skills over time among providers with different physical attributes could provide insights into how these skills can be honed and adapted.

## Conclusions

In conclusion, our study highlights the impact of hand size on the effectiveness of different FMV techniques, demonstrating that smaller-handed trainees achieved better ventilation with the VE technique compared to the CE technique. These findings have important implications for airway management training, suggesting that individualized instruction based on physical attributes may enhance skill acquisition and performance. As the demographics of healthcare providers continue to shift, with the increasing representation of women in anesthesiology and surgery, training programs should consider incorporating techniques adaptable to varying hand sizes and strengths. Additionally, future research should explore the role of other physical characteristics, such as thumb length and grip strength, in optimizing FMV techniques. Ultimately, ensuring inclusive training and technique modifications can help all providers achieve effective ventilation, improving patient safety in clinical settings.
